# Complementary Sex Determination in the Parasitic Wasp *Diachasmimorpha longicaudata*

**DOI:** 10.1371/journal.pone.0119619

**Published:** 2015-03-19

**Authors:** Leonela Carabajal Paladino, Irina Muntaabski, Silvia Lanzavecchia, Yoann Le Bagousse-Pinguet, Mariana Viscarret, Marianela Juri, Luciana Fueyo-Sánchez, Alba Papeschi, Jorge Cladera, María José Bressa

**Affiliations:** 1 Institute of Entomology, Biology Centre, Czech Academy of Sciences, Ceske Budejovice, Czech Republic; 2 Instituto de Genética “Ewald A Favret,” Instituto Nacional de Tecnología Agropecuaria, Hurlingham, Argentina; 3 Department of Botany, University of South Bohemia, Ceske Budejovice, Czech Republic; 4 Instituto de Microbiología y Zoología Agrícola, Instituto Nacional de Tecnología Agropecuaria, Hurlingham, Argentina; 5 Consejo Nacional de Investigaciones Científicas y Técnicas, Ciudad Autónoma de Buenos Aires, Argentina; 6 Instituto de Ecología y Desarrollo Sustentable, Universidad Nacional de Luján, Luján, Argentina; 7 Departamento de Ecología, Genética y Evolución, Facultad de Ciencias Exactas y Naturales, Universidad de Buenos Aires, Ciudad Autónoma de Buenos Aires, Argentina; 8 Instituto de Ecología, Genética y Evolución de Buenos Aires, Facultad de Ciencias Exactas y Naturales, Universidad de Buenos Aires, Ciudad Autónoma de Buenos Aires, Argentina; MRC Laboratory of Molecular Biology, UNITED KINGDOM

## Abstract

We studied the sex determination in *Diachasmimorpha longicaudata*, a parasitoid braconid wasp widely used as biological control agent of fruit pest tephritid flies. We tested the complementary sex determination hypothesis (CSD) known in at least 60 species of Hymenoptera. According to CSD, male or female development depends on the allelic composition of one sex locus (single-locus CSD) or multiple sex loci (multiple-locus CSD). Hemizygote individuals are normal haploid males, and heterozygotes for at least one sex locus are normal diploid females, but homozygotes for all the sex loci are diploid males. In order to force the occurrence of diploid males in *D*. *longicaudata*, we established highly inbred lines and examined their offspring using chromosome counting, flow cytometry, and sex ratio analysis. We found that when mother-son crosses were studied, this wasp produced about 20% of diploid males out of the total male progeny. Our results suggest that this parasitoid may represent the second genus with multiple-locus CSD in Hymenoptera. Knowledge about the sex determination system in *D*. *longicaudata* is relevant for the improvement of mass rearing protocols of this species. This information also provides the necessary background for further investigations on the underlying molecular mechanisms of sex determination in this species, and a better insight into the evolution of this pathway in Hymenoptera in particular and insects in general.

## Introduction

Sex determination refers to the developmental program which commits an individual to female or male path [1]. In Hymenoptera, the sex is determined by haplodiploidy (reviewed in [2, 3]). Even though this mechanism does not rely on the presence of sex chromosomes, males and females differ in their chromosome constitution [4]. Males are haploid, having only one set of maternally inherited chromosomes, and females are diploid, with both paternal and maternal chromosomes [1, 4, 5]. However, diploid males were described in 83 species [6], showing that sex determination in Hymenoptera is a more complex process. These males are typically found in situations where genetic diversity drastically decreases, as in inbred populations [7]. They can be viable or unviable, fertile or sterile [4, 7]. In any case, diploid males impose a genetic load on the population, bias the sex ratio in favour of males, and reduce the females’ reproductive potential as diploid males originate from fertilised eggs [4, 8].

The occurrence of diploid males does not fit into the mechanism of haplodiploidy as described above. New elements were, therefore, included in this model of sex determination and several derived models appeared: maternal-zygotic balance [9], genetic balance [10], genetic imprinting [9], fertilization sex determination [11], maternal effect sex determination [12], and complementary sex determination (CSD) or allelic diversity model, in one locus or multiple loci [13–17].

Under the CSD model the sex is determined by the state of a unique sex locus (single-locus CSD) or several sex loci (multiple-locus CSD). When hemizygous, the individual will develop into a haploid male, when heterozygous for at least one locus, the individual will be a diploid female, and when homozygous for the single sex locus or for all sex loci, the individual will become a diploid male [1, 5, 6]. From the population genetics point of view, the frequency of diploid males is mostly a function of the number of sex-determining alleles segregating in a given population, and of new alleles introduced by mutation. The CSD model may be numerically tested comparing sex ratios under inbreeding and under outbreeding, and analysing how often diploid males occur—they can be detected by cytogenetic methods (chromosome counting), flow cytometry, or molecular markers [4, 18, 19].

The CSD model was described in 60 hymenopteran species [6], and phylogenetic studies suggest that it could be an ancestral mechanism for Hymenoptera [5, 20, 21]. However, it is still unclear whether single-locus preceded multiple-locus CSD or vice versa [4, 21].

The aim of the present work is to analyse the presence of CSD in an economically important parasitoid wasp, *Diachasmimorpha longicaudata* (Ashmead) (Hymenoptera: Ichneumonoidea: Braconidae). This species is a solitary koinobiont endoparasitoid of tephritid fruit flies. It is widely used as a biological control agent of these pests [22] and massively produced in several countries of the American continent [23]. Knowledge about its physiology, behaviour, and genetics are required to develop better mass rearing protocols, which ensure and maximise the production of control agents of good quality and performance in nature [24]. An important aspect is to identify the sex determination system of this species, in order to put forward the measures necessary to increase female production, since only females cause damage to the immature stages of fruit flies pests in the field, and they determine the productivity in the bio-factory.

According to some authors [21] single-locus CSD should be an ancestral sex determination mechanism for Ichneumonoidea. In order to test the validity of the CSD model in *D*. *longicaudata*, we conducted a series of experiments that increased the inbreeding conditions to its maximum, hence forcing the occurrence of diploid males. Then, we used sex ratio analysis, cytogenetic techniques and flow cytometry to evaluate the presence of diploid males in the populations obtained. We also carried out a cytological study of diploid males in order to infer their reproductive potential. Our results provide useful information for improving the mass production of this important biological control agent and for further study sex determination in the order Hymenoptera.

## Materials and Methods

### Insects

The parasitoid *Diachasmimorpha longicaudata* and its host, the fruit fly *Ceratitis capitata* (Wiedemann) (Diptera: Tephritidae), were obtained from the experimental rearing facility established at the Instituto de Genética, INTA, Hurlingham, Buenos Aires, Argentina [25]. Adult *D*. *longicaudata* were originally imported from México to Tucumán province (Argentina) in 1998 (SENASA, exp. N° 14054/98) and were introduced to the Instituto de Genética in 2001. By the time the experiments were carried out, they had been reared under artificial conditions for ca. 100 generations. Adult parasitoids were maintained in glass flasks (volume 3 L) with water and honey at a density of ca. 20 individuals per litre. Larvae of *C*. *capitata* were reared on artificial larval diet [26] and when reaching the third larval instar, they were offered to adult *D*. *longicaudata* females. The larvae were confined in plastic Petri dishes (5 cm in diameter and 1 cm in depth) covered with voile fabric and exposed to the female wasps for a period of 4 h. Then, the larvae were transferred into a plastic tray with fresh artificial larval diet, where they completed their development. The trays were placed over a thin layer of vermiculite, used as pupation substrate. The individuals of both species were maintained in an incubator under controlled conditions (25°C, RH 85%, 18:6 h light:dark cycle) throughout all the experiments.

### Establishment of inbred lines by mother-son crosses

Forty four virgin 7-day-old *D*. *longicaudata* females were individually offered *ad libitum C*. *capitata* larvae confined in round metal containers (1.5 cm in diameter and 0.5 cm in depth) wrapped with voile, during an exposition time of 4 h. This period length is routinely applied in our laboratory for the rearing of *D*. *longicaudata* in order to minimise the possibilities of superparasitism, or death of the host due to confinement in the oviposition unit. After this time, each group of larvae were transferred into plastic cups with fresh artificial larval medium and placed in a glass flask (volume 400 mL) with a lid of voile cloth and a thin layer of vermiculite in the bottom. Larvae as well as pupae were maintained under the environmental conditions described above. After the *D*. *longicaudata* male progeny emerged, one male was randomly chosen from each flask and mated to his mother. Two days later, *C*. *capitata* larvae were offered to each parasitoid female following the procedure previously described.

### Experiments

The 44 isofemale lines were randomly divided into three groups assigned to the following experiments: (i) sex ratio analysis in adult stage (14 lines), (ii) cytogenetic and flow cytometry studies (15 lines each), and (iii) cytological analysis (data were obtained from the 15 lines assigned to the cytogenetic study).

#### (i) Sex ratio analysis in adult stage

**Fig 1:**
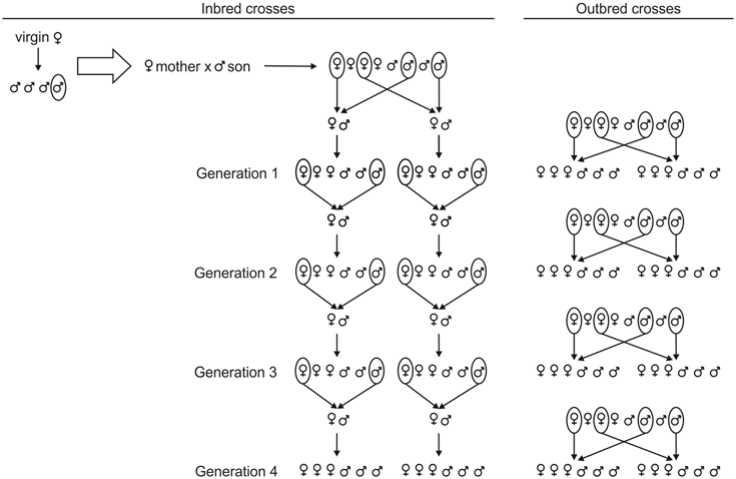
Experimental crosses. Schematic representation of the crosses performed in each generation of the isofemale lines and their respective exogamic controls.

Pupae of each virgin female-son cross (generation 0, G_0_) were isolated in order to obtain virgin females and males. For each inbred line, two males and two females were randomly selected and used to create sib-matings, resulting therefore in two replicates per G_0_ mother. This procedure was repeated for four generations (G_1_ to G_4_), but performing only one random brother-sister cross, as to maintain a steady number of lines (14) and replicates per line (two) (Fig. 1). Ten exogamic control crosses were carried out per generation using virgin females and non-related males obtained from the artificial rearing kept in our laboratory (Fig. 1). Two expositions of *C*. *capitata* larvae were performed in consecutive days, and the data obtained from both experiments were pooled together. In each generation the number of emerged female and male parasitoids, the number of non-emerged fly puparia and the number of emerged flies were recorded. The sex ratio was then estimated as the proportion of males (number of males / total number of parasitoids in the progeny).

#### (ii) Cytogenetic and flow cytometry studies

*Cytogenetic study*. Fly puparia from the mother-son crosses were dissected 11 to 13 days after parasitoidisation. The immature parasitoids recovered from the fly puparia were dissected under a stereoscopic microscope Olympus SZ-11 (Olympus Corporation, Tokyo, Japan). Their sex was determined externally by the presence of an ovipositor in females, and the valves that constitute the external part of the reproductive system in males, or internally by the presence of ovaries or testes [27]. Third instar male larvae, prepupae and pupae, were used for making chromosome preparations by spreading testes according to [28] with slight modifications described in [29]. The preparations were then dehydrated in an ethanol series (70%, 80%, and 96%, 30 s each), inspected in phase contrast and after staining (see below) photographed using a Leica DMLB microscope equipped with a Leica DFC 350 FX CCD camera and Leica IM50 Software, version 4.0 (Leica Microsystems Imaging Solutions Ltd., Cambridge, UK).

For detailed analysis, the chromosome preparations were stained with 0.5 μg/mL DAPI (4’,6-diamidino-2-phenylindole; Fluka BioChemika, Sigma Aldrich Production GmbH, Buchs, Switzerland) in PBS containing 1% Triton X-100, and mounted in Vectashield Mounting Medium (Vector Laboratories, Burlingame, CA, USA). The chromosome preparations were observed under the Leica DMLB microscope. Black-and-white images of ten to 20 metaphases per individual were recorded and processed with appropriate software. Ploidy level of each male was determined by chromosome counting, n = 20 chromosomes for haploid individuals and 2n = 40 chromosomes for diploid individuals [30, 31]. The total amount of diploid and haploid males and females obtained per line was recorded.

**Fig 2:**
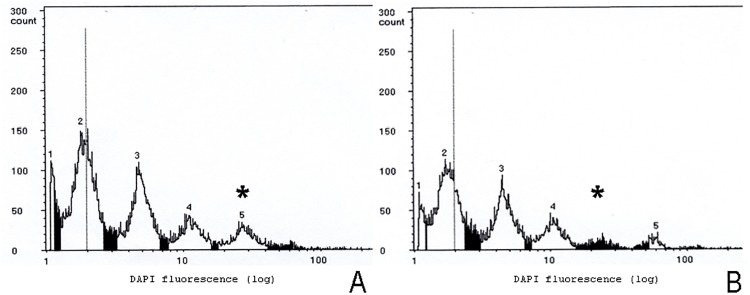
Flow cytometry profiles from *D*. *longicaudata* control individuals. (A) Diploid (virgin) female. (B) Haploid male (son of virgin female). Asterisk indicates the differential peak.

*Flow cytometry study*. The offspring of mother-son crosses was allowed to emerge. Males were analysed by flow cytometry to measure their ploidy level. Due to the small size of *D*. *longicaudata* and restrictions inherent to the equipment available for the analysis, the entire body of each individual instead of just the head was used for the experiments. As endoduplication (replication of nuclear genome in the absence of cell division) occurs in different body parts, no haploid and diploid peaks could be identified, alternatively, a haploid and diploid histogram profile was obtained. Before each set of measurements, we studied one virgin female and one of its sons in order to identify the diploid and haploid patterns of peaks (Fig. 2). Each individual was chopped with a sharp razor blade in 500 μL of nuclei extraction buffer, and the suspension containing the released nuclei was passed through a 30 μm filter. Then, the nuclei in filtrate suspension were stained with 1 mL of solution containing DAPI [32]. After shaking the solution gently, samples were analysed with a flow cytometer (PA Ploidy Analyser, Partec, Münster, Germany). Ploidy level of the experimental males was determined by comparison with the pattern of peaks observed in the control individuals after analysing at least 10,000 events. The number of haploid and diploid males and females was recorded.

#### (iii) Cytological analysis

The photographs taken for chromosome counting were used to measure the nuclear area of the spermatocytes of nine diploid males and nine haploid males (n = 419 measurements in each group). The measurements were carried out using software JMicroVision v1.2.7 [33]. Calibration was performed using image resolution hence, the area of each nucleus was measured in pixels. The average nucleus size of each male was calculated.

### Statistical analyses

#### i) Sex ratio analysis in adult stage

The sex ratio was analysed using a global linear mixed effect model with generation (G_1_ to G_4_), treatment (inbreeding or outbreeding) and their interaction as predictors, and the proportion of males as the response variable. The family was included as random factor. Considering that there might be differences in the mortality of diploid and haploid males, the proportion of males was also analysed considering the mortality rate [non-emerged puparia / (non-emerged puparia + emerged parasitoids of both sexes + emerged flies)] as random factor. Moreover, the same statistical model used for analysing the sex ratio was applied with the mortality rate as the response variable, in order to corroborate any differences in the mortality of diploid and haploid males due to the inbreeding or outbreeding treatment.

#### ii) Cytogenetic and flow cytometry studies

In both experiments it was not possible to determine the ploidy level of some males. One possible explanation is premature death or problems during the development of diploid males hence, the proportion of diploid males was calculated considering two extreme situations: (a) excluding these males from the analysis [diploid males / (diploid males + females)], or (b) including them as diploid males [(diploid males + males of unknown ploidy) / (diploid males + males of unknown ploidy + females)].

In order to test differences between methods to detect diploid males, cytogenetic and flow cytometry were considered fixed factors in a one way ANOVA model. Excluding from the analysis all the males of unknown ploidy, no statistically significant difference between methods was detected (*F*_(1; 25)_ = 3.604, *p* = 0.07), the data were pooled and pairwise sample *t*-tests were used to compare the observed proportion of diploid individuals that are males with the expected under CSD model with one locus (0.5), two loci (0.25), three loci (0.125) and four loci (0.0625). The expected frequencies consider: (a) independent segregation of two sex alleles per locus, as the use of virgin mothers with one of their sons limits the quantity of sex alleles to that number; and (b) equal probability of fertilization of the spermatozoa carrying the different alleles or allele combination.

Including as diploid males all the males of unknown ploidy, statistically a significant difference between methods was detected (*F*_(1; 25)_ = 6.0897, *p* = 0.02); and the pairwise sample *t*-tests were performed for each method independently.

#### iii) Cytological analysis

The average nucleus size was compared between haploid and diploid males (fixed factor) using a one way ANOVA model.

All statistical analyses were done using R Core Team 2.15.1 [34].

## Results

### i) Sex ratio analysis in adult stage

The statistical analysis of the proportion of males showed a negligible effect of the family on the observed variances (Standard deviation = 3.94.10^-6^), and no statistically significant effect of the interaction between treatment and generation (df = 3; χ^2^ = 0.63; *p* = 0.889). There was a statistically significant effect of the inbreeding treatment (df = 1; χ^2^ = 8.167; *p* = 0.004) generating more males; and of the generation (df = 3; χ^2^ = 12.27; *p* = 0.007), showing an increase in the number of males produced through time. We detected no significant effect of mortality on the sex ratio (Standard deviation = 6.093.10^-6^).

**Fig 3:**
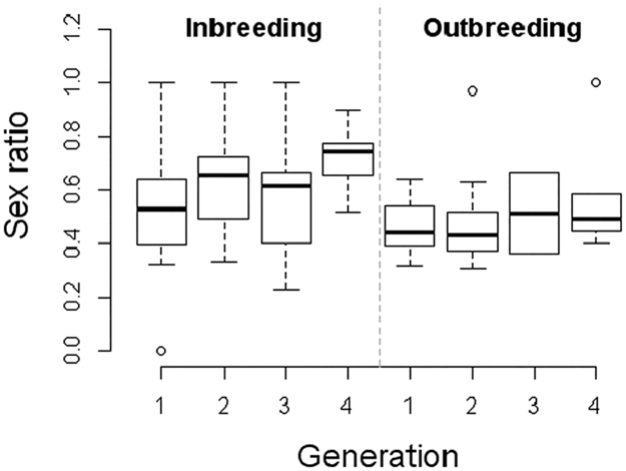
Sex ratio analysis. Sex ratio values representation for the inbreeding and outbreeding treatments in each of the four generations that were analysed. Lower hinge = 25^th^ percentile, higher hinge = 75^th^ percentile, segment inside the box = median (50^th^ percentile), whiskers = maximum and minimum values, unfilled circle = outside value.

The statistical analysis of the mortality rate showed a negligible effect of the family (Standard deviation = 0.02), and no statistically significant effect of the interaction between treatment and generation (df = 3; χ^2^ = 0.63; *p* = 0.06). There was no statistically significant effect of the inbreeding treatment (df = 1; χ^2^ = 3.382; *p* = 0.07); but there was an effect of the generation (df = 3; χ^2^ = 33.424; *p* < 0.01), probably due to the high variance of the measurements observed in G_3_ (Table 1). Note that in G_3_, only two out of the ten exogamic couples produced offspring; thereby an artifactual variance was observed in that point (Fig. 3). Also, during the four generations a total of nine inbred replicates were lost due to production of only male or only female offspring, or death of the female before oviposition, making it impossible to form sibling couples (Table 1).

**Table 1:** Number of females, males, non-emerged pupae, and flies obtained in the inbred and outbred lines during four generations.

	Family	G_1_						G_2_						G_3_						G_4_					
		F	M	S. R.	Non-E.	Flies	Mort.	F	M	S. R.	Non-E.	Flies	Mort.	F	M	S. R.	Non-E.	Flies	Mort.	F	M	S. R.	Non-E.	Flies	Mort.
Inbred crosses	1A	18	20	0.53	15	124	0.08	11	23	0.68	13	127	0.07	5	9	0.64	7	34	0.13	2	18	0.90	17	226	0.06
	1B	12	12	0.50	10	82	0.09	20	10	0.33	15	123	0.09	6	4	0.40	7	39	0.13	19	26	0.58	13	227	0.05
	2A	11	26	0.70	21	115	0.12	8	21	0.72	9	67	0.09	11	11	0.50	8	42	0.11	13	38	0.75	23	248	0.07
	2B	0	51	**1**	14	88	0.09	-	-	-	-	-	-	-	-	-	-	-	-	-	-	-	-	-	-
	3A	15	14	0.48	12	85	0.10	0	15	**1**	8	112	0.06	-	-	-	-	-	-	-	-	-	-	-	-
	3B	24	14	0.37	18	163	0.08	3	7	0.70	6	143	0.04	13	8	0.38	9	64	0.10	20	27	0.57	17	153	0.08
	4A	32	19	0.37	34	147	0.15	0	24	**1**	12	121	0.08	-	-	-	-	-	-	-	-	-	-	-	-
	4B	5	0	**0**	39	141	0.21	-	-	-	-	-	-	-	-	-	-	-	-	-	-	-	-	-	-
	5A	9	11	0.55	28	68	0.24	6	24	0.80	19	142	0.10	5	10	0.67	15	73	0.15	Female died before oviposition					
	5B	19	10	0.34	12	119	0.08	11	12	0.52	8	95	0.06	5	3	0.38	11	62	0.14	15	45	0.75	38	214	0.12
	6A	20	16	0.44	34	113	0.19	10	30	0.75	23	137	0.12	12	5	0.29	14	34	0.22	13	25	0.66	34	192	0.13
	6B	13	15	0.54	16	84	0.13	8	12	0.60	12	84	0.10	3	5	0.63	10	34	0.19	15	23	0.61	30	189	0.12
	7A	9	6	0.40	10	91	0.09	22	15	0.41	11	77	0.09	10	3	0.23	8	38	0.14	17	33	0.66	34	358	0.08
	7B	9	14	0.61	36	122	0.20	10	21	0.68	14	95	0.10	6	17	0.74	8	39	0.11	5	20	0.80	35	173	0.15
	8A	25	12	0.32	14	91	0.10	23	15	0.39	8	123	0.05	1	2	0.67	7	53	0.11	8	16	0.67	25	288	0.07
	8B	4	32	0.89	12	110	0.08	7	13	0.65	8	69	0.08	1	10	0.91	7	84	0.07	0	0	**n/a**	16	311	0.05
	9A	10	27	0.73	23	160	0.10	12	12	0.50	27	92	0.19	1	8	0.89	14	52	0.19	19	23	0.55	21	254	0.07
	9B	14	9	0.39	16	104	0.11	6	7	0.54	11	135	0.07	3	4	0.57	5	20	0.16	9	34	0.79	31	277	0.09
	10A	17	16	0.48	22	93	0.15	7	18	0.72	5	95	0.04	8	9	0.53	13	76	0.12	13	14	0.52	40	230	0.13
	10B	2	3	0.60	15	134	0.10	19	18	0.49	11	117	0.07	0	0	**n/a**	2	54	0.04	-	-	-	-	-	-
	11A	15	20	0.57	19	97	0.13	7	33	0.83	11	85	0.08	1	6	0.86	8	71	0.09	11	37	0.77	13	206	0.05
	11B	24	14	0.37	20	102	0.13	4	8	0.67	12	104	0.09	0	14	**1**	4	29	0.09	-	-	-	-	-	-
	12A	3	9	0.75	6	108	0.05	8	15	0.65	18	97	0.13	5	8	0.62	14	58	0.16	5	25	0.83	20	163	0.09
	12B	7	17	0.71	22	116	0.14	5	10	0.67	11	118	0.08	5	9	0.64	9	23	0.20	10	34	0.77	24	275	0.07
	14A	12	11	0.48	29	122	0.17	12	9	0.43	17	76	0.15	18	9	0.33	18	51	0.19	8	27	0.77	11	265	0.04
	14B	9	12	0.57	29	155	0.14	22	16	0.42	21	142	0.10	5	6	0.55	8	67	0.09	11	20	0.65	38	301	0.10
	15A	15	27	0.64	20	86	0.14	0	0	**n/a**	6	132	0.04	-	-	-	-	-	-	-	-	-	-	-	-
	15B	5	9	0.64	28	106	0.19	0	0	**n/a**	20	108	0.16	-	-	-	-	-	-	-	-	-	-	-	-
Outbred crosses	1	12	14	0.54	16	144	0.09	14	15	0.52	9	146	0.05	7	4	0.36	5	53	0.07	24	16	0.40	29	259	0.09
	2	13	10	0.43	14	134	0.08	12	8	0.40	14	119	0.09	0	0	**n/a**	1	80	0.01	21	17	0.45	31	157	0.14
	3	28	13	0.32	9	96	0.06	15	13	0.46	1	90	0.01	0	0	**n/a**	2	74	0.03	0	39	1.00	30	207	0.11
	4	21	17	0.45	19	82	0.14	23	14	0.38	6	94	0.04	0	0	**n/a**	3	99	0.03	38	34	0.47	27	253	0.08
	5	14	14	0.50	8	125	0.05	7	12	0.63	15	113	0.10	0	0	**n/a**	1	62	0.02	16	19	0.54	10	168	0.05
	6	27	19	0.41	12	124	0.07	26	17	0.40	13	146	0.06	0	0	**n/a**	6	95	0.06	35	25	0.42	18	254	0.05
	7	7	10	0.59	6	71	0.06	33	18	0.35	14	138	0.07	2	4	0.67	4	30	0.10	0	47	1	16	328	0.04
	8	5	9	0.64	4	110	0.03	1	31	0.97	9	93	0.07	0	0	**n/a**	0	0	**n/a**	25	26	0.51	15	200	0.06
	9	23	14	0.38	12	158	0.06	12	12	0.50	13	146	0.07	0	0	**n/a**	0	75	0	31	27	0.47	26	181	0.10
	10	22	14	0.39	9	97	0.06	9	4	0.31	5	98	0.04	0	0	**n/a**	5	64	0.07	22	31	0.58	16	216	0.06

G_1_ = Generation 1, G_2_ = Generation 2, G_3_ = Generation 3, G_4_ = Generation 4, A–B = replicates within each inbred family, F = females, M = males, S. R. = sex ratio, Non-E. = non-emerged pupae, Mort. = mortality rate, n/a = not applicable.

### ii) Cytogenetic and flow cytometry studies

**Fig 4:**
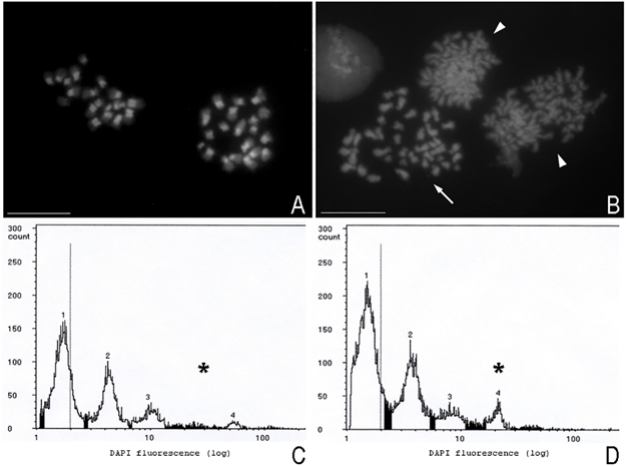
Chromosome constitution and flow cytometry profiles of haploid and diploid experimental males. (A–B) DAPI-stained chromosome preparations obtained by spreading from gonads of male descendants of mother–son crosses. Bar = 10 μm. (A) Haploid male metaphase with n = 20 chromosomes. (B) Diploid male metaphase with n = 40 chromosomes (arrow) and two anaphases (arrow heads). (C–D) Flow cytometry profiles from *D*. *longicaudata* experimental individuals. (C) Haploid male. (D) Diploid male. Asterisk indicates the differential peak.

Males in larval, prepupa and pupa stages with a haploid (n = 20 chromosomes) and a diploid (2n = 40 chromosomes) chromosome number were found in *D*. *longicaudata* under inbreeding conditions (Fig. 4A, B). Ten crosses produced descendants of both sexes, indicating that the female was fertilised. Data from these families were taken into account for further analysis. In total, 84 females, 108 males, and 55 individuals of uncertain sex were recorded (Table 2).

**Table 2:** Sex and ploidy level of the offspring of mother–son crosses analysed by cytogenetic techniques and flow cytometry.

Method	Line	Sex (?)	Females	Males			Prop a	Prop b
				Haploid	Diploid	Ploidy (?)		
Cytogenetics	2	2	12	10	2	3	0.143	0.294
	4	5	13	8	4	9	0.235	0.500
	5	7	10	6	0	7	0	0.412
	6	4	6	6	1	2	0.143	0.333
	8	3	3	5	0	0	0	0
	11	10	5	5	0	3	0	0.375
	12	5	7	3	0	3	0	0.300
	13	5	9	7	1	7	0.100	0.471
	14	6	10	1	2	0	0.167	0.167
	15	8	9	7	5	1	0.357	0.400
Total		55	84	58	15	35		
Flow cytometry	1	16	3	5	2	0	0.400	0.400
	2	17	9	7	0	10	0	0.526
	3	14	13	13	3	7	0.188	0.435
	4	13	15	9	0	6	0	0.286
	5	12	10	6	0	7	0	0.412
	6	2	2	11	2	2	0.500	0.667
	7	7	10	9	2	2	0.167	0.286
	8	6	1	8	1	2	0.500	0.750
	9	9	6	14	1	5	0.143	0.500
	10	14	1	8	1	2	0.500	0.750
	11	8	8	15	2	9	0.200	0.579
	12	10	10	9	1	7	0.091	0.444
	13	8	15	15	6	3	0.286	0.375
	14	19	6	7	3	2	0.333	0.455
	15	3	14	6	5	1	0.263	0.300
Total		158	123	142	29	65		

“(?)” indicates uncertainty.

Prop a = proportion of diploid individuals that are males excluding the males of unknown ploidy, Prop b = proportion of diploid individuals that are males including the males of unknown ploidy as diploid males.

Three to 21 males were karyotyped in each inbred line. A total of 108 male chromosome preparations were analysed, out of them 49 corresponded to haploid males (n = 20 chromosomes) (Fig. 4A), six corresponded to diploid males (2n = 40 chromosomes) (Fig. 4B), nine were classified as haploid males but with less certainty since an insufficient number of chromosome metaphases was observed, and nine were classified as diploid males also with less certainty. The results show that about 20% of the immature males that originated from a mother–son cross were diploid. These individuals represent about 15% of the diploid offspring (i.e. [diploid males / (diploid males + females)]*100). In 35 out of the 108 analysed males, no cells in metaphase stage were found, making impossible to determine their ploidy level.

Haploid and diploid adult males were identified by flow cytometry in *D*. *longicaudata* under inbreeding conditions (Fig. 4C, D). All 15 crosses produced offspring of both sexes. A total of 171 males were analysed by flow cytometry, 142 being haploid (Fig. 4C) and the remaining 29 being diploid (Fig. 4D) (Table 2). The results show that about 17% of the adult male progeny from a mother–son cross were diploid males. These individuals represent about 19% of the diploid offspring. The ploidy level of 65 individuals could not be assessed as they died before the analysis could be carried out, and no accurate profile could be obtained from them.

The comparison of the observed proportion of diploid individuals that are males with the expected values under CSD using different number of loci showed that the frequency of diploid males fits more closely to a model of complementary allele sex determination with one to three independent loci (Table 3).

**Table 3:** *p*-values obtained from the pairwise sample *t*-test when comparing the observed proportion of diploid individuals that are males, with the values expected under CSD with one locus, two loci, three loci and four loci.

Proportion of diploid individuals that are males (excluding the males of unknown ploidy)					
Method	Observed	Expected			
		One locus	Two loci	Three loci	Four loci
Cytogenetics + Flow cytometry	0.1886	0.5	0.25	0.125	0.0625
		*p* < 0.0001	***p* = 0.079**	***p* = 0.07**	*p* < 0.0001

Proportion of diploid individuals that are males (including the males of unknown ploidy as diploid males)					
Method	Observed	Expected			
		One locus	Two loci	Three loci	Four loci
Cytogenetics	0.3251	0.5	0.25	0.125	0.0625
		*p* < 0.005	***p* = 0.1452**	*p* < 0.005	*p* < 0.0005
Flow cytometry	0.5011	0.5	0.25	0.125	0.0625
		***p* = 0.9844**	*p* < 0.005	*p* < 0.0001	*p* < 0.0001

### iii) Cytological analysis

**Fig 5:**
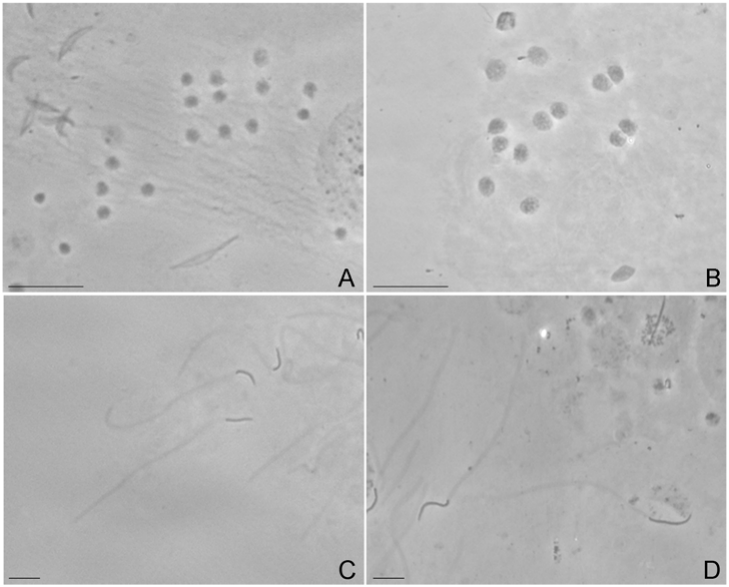
Spermatocytes and spermatozoa of haploid and diploid males. Phase-contrast images of chromosome preparations obtained by spreading of gonads from male descendants of mother–son crosses. (A) Haploid spermatocytes. (B) Diploid spermatocytes. (C) Haploid spermatozoa. (D) Diploid spermatozoa. Bar = 10 μm.

The analysis of chromosome preparations showed that both haploid and diploid males produce spermatids and mature spermatozoa (Fig. 5). Haploid (Fig. 5A) and diploid (Fig. 5B) males showed significant differences in the mean nuclear area of their spermatocytes (*F*_(1, 18)_ = 6.038; *p* = 0.0258). The mean (±S.E.) was 12.40±1.47 pixels and 8.65±0.42 pixels, for diploid and haploid male spermatocytes, respectively. Spermatozoa from haploid and diploid males had similar morphology, and only differed in their size (Fig. 5C, D).

## Discussion

Our study represents the first attempt to elucidate the sex determination system in *D*. *longicaudata*, and provides the first description of diploid males in this species using both direct (chromosome counting and flow cytometry profile) and indirect (secondary sex ratio analysis) methods. The experiments of sex ratio with inbred lines showed an increase in the proportion of males across inbreeding generations. These differences in the sex ratio together with the lack of differences in the mortality rate between treatments, suggest the production of viable diploid males, and are consistent with the possible existence of multiple-locus CSD in this species. The results obtained by chromosome counting and flow cytometry suggest a number of sex determining loci ranging from one to three.

**Fig 6:**
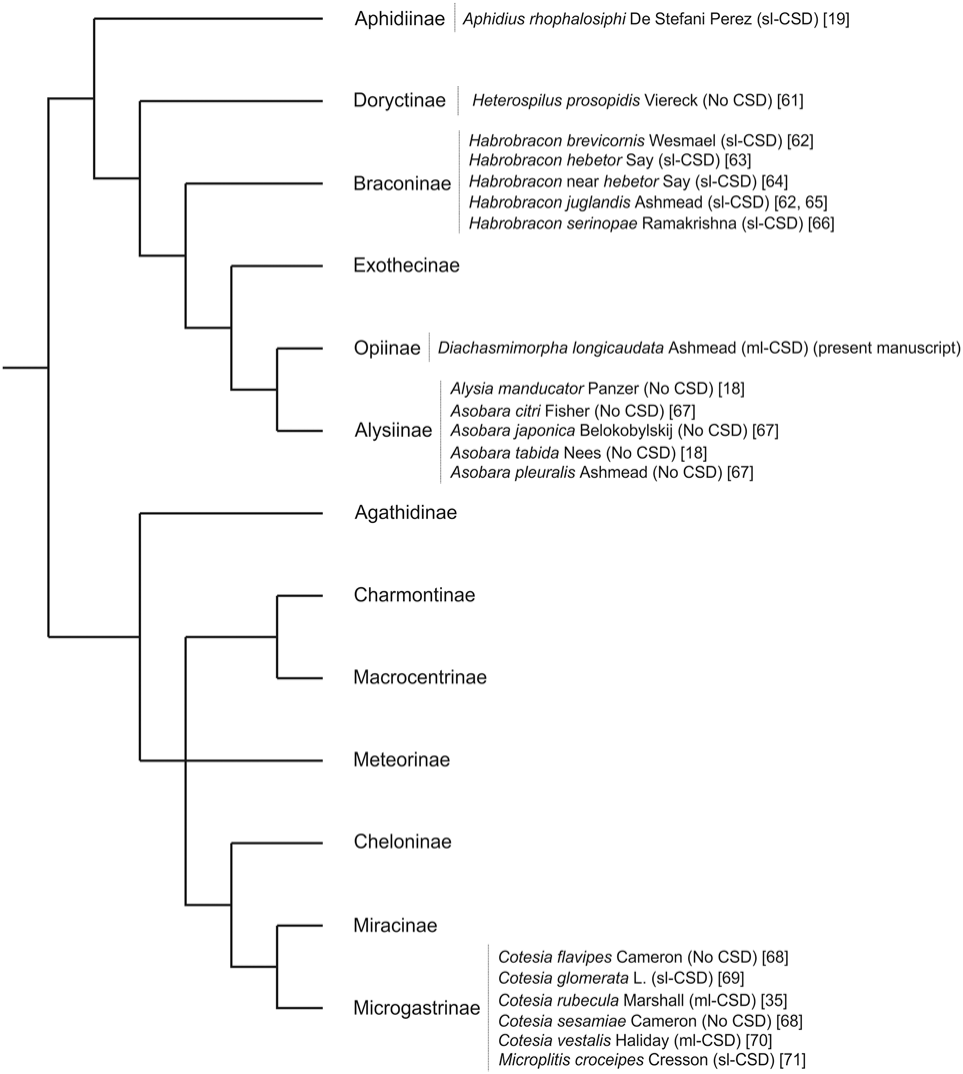
Distribution of CSD in Braconidae. Phylogeny of the family Braconidae (from [59]; simplified from [60]). Species so far analysed for complementary sex determination (CSD) are included [18, 19, 35, 61–71]. sl-CSD = single-locus CSD, ml-CSD = multiple-locus CSD.

Multiple-locus CSD has only been experimentally proved in the braconids *Cotesia vestalis* (Haliday) [7] and *C*. *rubecula* (Marshall) [35]. From 19 braconid species known to produce diploid males (including *D*. *longicaudata*), three show multiple-locus CSD, eight have single-locus CSD, and the CSD model was ruled out in the rest (Fig. 6). Furthermore, five out of the eight species without CSD belong to the subfamily Alysiinae, sister subfamily of Opiinae, to which *D*. *longicaudata* belongs. Hence, our results call for a deeper study of the intra-taxon variation of sex determination systems, and stress the importance of the analysis of this trait in the order Hymenoptera.

Population genetics of the *D*. *longicaudata* strain used in these experiments should be considered. The population under study has been kept under artificial rearing during ca. 100 generations without being refreshed with wild insects [26], and has suffered several bottleneck episodes throughout its maintenance in laboratory conditions. It is therefore plausible for some sex loci to be fixed in the homozygous state, causing a reduction in complexity of the sex determination system (from more to less sex loci) [3, 35]. This may explain the variation observed between experiments in the estimation of diploid males frequency, and then in the detection of one locus to three loci involved in sex determination. In addition, the CSD model states that an individual will be female if at least one of the sex loci is in the heterozygous state. However, in this study it was not possible to accurately determine the number of heterozygous loci in the females used during the experiments. Hence, we estimated a minimum number of sex loci (one to three) involved in the sex determination of this species.

It is worth noticing that no obvious changes from the 1:1 sex ratio were observed in the laboratory colony of *D*. *longicaudata* kept at the Instituto de Genética, INTA (MC Maninno, pers. comm.), and that during several years of cytogenetic studies no diploid males were detected [31], suggesting that *D*. *longicaudata* has developed adaptations to reduce the negative impacts of diploid male production, as described for other species forced to high inbreeding [3, 4, 20, 36]. For insect populations in the wild these adaptations include for instance, differential developmental rates between sexes, which may allow pre-mating dispersal of the individuals and thus reduce the risk of sib-mating [20, 37, 38]. Actually, *D*. *longicaudata* males emerge two days before females [27]. However, this has no impact on the artificial rearing, as all siblings are kept in the same cage. Hence, other genetic mechanisms of inbreeding avoidance, such as high rate of recombination in specific loci in the genome [39–41] and behavioural pre- and/or post-copulatory traits like paternity biasing by polyandrous females [42–44], kin discrimination during male choice [45, 46], active female choice of sperm [47–49], or male-male or sperm competition favouring unmatched mates [50] should be further investigated in *D*. *longicaudata*.

The analysis of the nuclear area of spermatocytes and the observation of spermatozoa in diploid males revealed that the diploid males can produce not only mature gametes, but that these gametes are normal in appearance. In *D*. *longicaudata*, spermatogenesis is characterised by the occurrence of an abnormal meiosis, similar to mitosis, with an abortive first meiotic division [51]. The larger size of the spermatocytes indicates that diploid males generate gametes in the same way, and without the chromosomal number reduction observed in other species, such as *Euodynerus foraminatus* (Saussure) (Hymenoptera, Vespidae) [52].

As *D*. *longicaudata* diploid males are viable, they could successfully copulate with females, contributing to mating failures if their sperm is not fertile. Previous studies in different hymenopteran species compared the reproductive behaviour, inseminating efficacy and fertility of diploid males with those of haploid males, showing that they may have similar characteristics (references in [6, 35]). Taking into account this information and the fact that *D*. *longicaudata* diploid males produce mature gametes, the ability of these males to successfully copulate with females and to fertilise their eggs deserves further research. This information is crucial to accurately determine the impact of diploid males in the population, as their production, if they are not fertile, could reduce population growth rates [53], effective population size [54], and increase extinction risk [55]. However, diploid male fertility may lower the costs associated with their production, thus sustaining persistent inbreeding, as previously reported for other species with functionally reproductive diploid males [52, 56–58].

The occurrence of diploid males presents a potential problem in terms of productivity levels of *D*. *longicaudata* strains maintained in mass-reared colonies for biological control strategies against fruit flies, and should therefore be accounted for [53]. Based on our study, mass-rearing protocols should try to maintain a high level of genetic variability to ensure heterozygosity at the sex loci. There are at least three ways by which a high heterozygosity can be sustained under artificial rearing: 1) initiating the colony with a large number of wild insects and keeping it large enough to ensure a high allele variability, 2) maintaining a large number of small populations, in which different alleles will be randomly fixed, but as a whole the variability of a larger size population will be preserved, 3) refreshing the colony with wild individuals [53].

In summary, we initiated the study of sex determination in *Diachasmimorpha longicaudata* using different approaches which constitute reliable ways to study sex determination in hymenopteran species. These were adequate to evidence the presence of viable diploid males and to propose a multiple-locus CSD system for this species. In addition, *D*. *longicaudata* was proven to be a valid and novel model organism for genetic and behavioural studies, as it can be easily handled and reared, and shows a high versatility for any kind of biological studies. The knowledge acquired about different genetic aspects of this species, such as the elucidation of a potential multiple-locus sex determination system, allows further investigations to be performed on the molecular basis of this trait, which is relevant not only for mass rearing of this and other braconid species of economic importance, but also for a better insight into the evolution of sex determination in Hymenoptera in particular and insects in general.
